# Insights into gait performance in Parkinson's disease via latent features of deep graph neural networks

**DOI:** 10.3389/fneur.2025.1567344

**Published:** 2025-06-20

**Authors:** Jiecheng Wu, Ning Su, Xinjin Li, Chao Yao, Jipeng Zhang, Xucheng Zhang, Wei Sun

**Affiliations:** ^1^Institute of Software, Chinese Academy of Sciences, Beijing, China; ^2^School of Computer Science and Technology, University of Chinese Academy of Sciences, Beijing, China; ^3^Peking Union Medical College Hospital, Beijing, China; ^4^The School of Computer and Communication Engineering, University of Science and Technology Beijing, Beijing, China; ^5^Health Service Department of the Guard Bureau of the Joint Staff Department, Beijing, China

**Keywords:** Parkinson's disease, gait analysis, quantitative assessment, graph convolutional network, skeleton-based data

## Abstract

**Introdcution:**

Parkinson's Disease (PD) is a progressive neurodegenerative disorder that primarily impacts motor function and is prevalent among older adults worldwide. Gait performance (such as speed, stride, step, and so on) has been shown to play a significant role in diagnosis, treatment, and rehabilitation. Fortunately, advancements in computer science have provided serial ways to calculate gait-related parameters, offering a more accurate alternative to the complex and often imprecise assessments traditionally relied upon by trained professionals. However, most of the current methods depend on data preprocessing and feature engineering, often require domain knowledge and laborious human involvement, and require additional manual adjustments when dealing with new tasks.

**Methods:**

To reduce the model's reliance on data preprocessing, feature engineering, and traversal rules, we employed the Spatial-Temporal Graph Convolutional Networks (ST-GCN) model. We also defined five distinct states within a complete gait cycle: standstill (S), left swing (L), double support (D), right swing (R), and turnaround (T). Using ST-GCN, we extracted spatial and temporal patterns from these five states directly from the data, thereby enhancing the accuracy of gait parameter calculation. Furthermore, to improve the interpretability of the ST-GCN model and increase its clinical relevance, we trained the model on data from both healthy individuals and PD patients. This allowed us to explore how the model's parameters (different ST-GCN Layers) could assist clinicians in understanding.

**Results:**

The dataset used to evaluate the model in this paper includes motion data from 65 PD participants and 77 healthy control participants. Regarding the classification results from the 5 classifiers, ST-GCN achieved an average precision, recall, and F1-score of 93.48%, 93.21%, and 93.32%, outperforming both the Transformer-based and LSTM-based methods. Displaying the joints and edge weights from various layers of the ST-GCN, particularly when comparing data from healthy individuals and PD patients, enhances the model's feasibility and offers greater interpretability. This approach is more informative than relying on a purely black-box model.

**Conclusion:**

This study demonstrated that the ST-GCN approach can effectively support accurate gait parameter assessment, assisting medical professionals in making diagnoses and reasonable rehabilitation plans for patients with PD.

## 1 Introduction

Parkinson's disease (PD) is one of the most common neurodegenerative diseases among people aged 60 and above (1). A variety of motor symptoms are common in Parkinson's disease, including impaired motor function with slow movements, tremors, and gait and balance disturbances (2). Gait disorder is one of the most common motor symptoms of Parkinson's disease (3). Its characteristic patterns include reduced turning agility, short and slow steps, festination, and freezing of gait (FoG), which generally occurs in patients with mid and advanced-stage PD (4). Considering the strong correlation between gait disorders and decreased quality of life, early recognition of gait disorders is crucial for the diagnosis, treatment, and prognosis of patients with PD (5). However, due to subtle and imperceptible changes in gait, it is difficult for neurologists and physicians to assess gait disorders in PD (6). In clinical practice, traditional scales are commonly used to assess gait disorders in PD, such as the Part III of the MDS-sponsored revision of the Unified Parkinson's Disease Rating Scale (MDS-UPDRS III) (7), the Timed Up and Go test (TUG) (8), the Freezing of Gait Questionnaire (FOG-Q) (9) and so on (10). Specifically, the physician must observe the patient's walking performance and then give a score based on the criteria of the scales.

Although traditional scales are widely used in clinical practice, they still have some limitations. Due to its semi-quantitative and time-consuming subjective evaluations of clinical physicians, the results may be imprecise or inconsistent, especially for PD with subtle symptoms. In recent years, along with the rapid development of science and technology, objective and quantitative gait assessment techniques have emerged (11). Instrumented gait analysis (IGA) uses motion capture systems and instrumented walkways to record gait data such as positions and pressures. Its accurate and precise quantitative measurement of gait performance makes it the gold standard for gait assessment in research practice (12). Quantitative assessment based on IGA can improve the diagnosis, prediction of outcome and rehabilitation of various gait disorders compared to conventional observational scales and techniques for gait disorders in PD. The variety of human gait parameters it provides, including spatio-temporal, kinematic, and kinetic parameters, can help researchers better identify and understand gait disorders (10). However, deploying IGA in clinical settings still faces various challenges. For example, vision-based IGA systems use multicameras to offer the highest capture accuracy, yet they are expensive and demand a large space for deployment, making them difficult to popularize currently. In addition, wearable sensor-based systems use wearable sensors such as inertial measurement units (IMUs) (13) and press the force insole to capture acceleration or pressure instead of video. They have both usability issues and technical challenges, such as discomfort during wear, data alignment, and bias drift. Meanwhile, depth sensors such as Kinect (14) are becoming popular in gait analysis. Their low-cost and non-contact advantages make them convenient and feasible for clinical deployment. What is more important, they not only provide depth video, but also offer human 3D skeleton data. This type of data represents the spatial positions of the main joint points of the human body, making it easier and more effective to describe human movements. Therefore, using a depth sensor is a promising method to assess gait performance. In addition to advances in these motion capture devices, significant progress has also been made in software algorithms, propelling PD gait research into a new stage.

The researchers relied mainly on hand-designed feature extraction methods to process skeleton data to assess gait disorders. These methods commonly required specialized knowledge and experience to select and design features. Researchers usually shape skeleton data into pseudoimages or coordinate vector sequences for machine learning methods. Procházka et al. (15) proposed a Bayesian system that used stride length, gait speed, and age as features. Then, digital signal processing methods and Bayesian probability classification algorithms were used for gait feature analysis to recognize individuals suspected of having Parkinson's disease. These methods are unsatisfactory because they cannot fully express the complex information contained in the skeleton data. With the rise of deep learning technology, several deep learning models have been applied to human motion analysis in skeleton data, such as Recurrent Neural Networks (RNNs) (16) and Long Short-Term Memory (LSTM) (17). These models construct the coordinates of the joint point into vector sequences that are used as input. Although these deep learning models make it easier to assess the severity of PD, they have the problem of lack of interpretability. Physicians and patients do not understand how these deep learning models make their predictions. A feasible solution is to apply deep learning models in the process of obtaining gait parameters. The key of the process is the detection of the gait phase. Jing et al. (18) used bidirectional Long Short-Term Memory (Bi-LSTM) to identify gait types in each walking frame. The results indicated that the Bi-LSTM model is better than the traditional machine learning method, Support Vector Machine (SVM). However, the ability of these models is relatively limited because they do not effectively utilize the spatial relationships between skeleton points, which are crucial to understanding human actions. In recent years, graph convolutional networks (GCNs) (19) have shown good performance in skeleton-based human action recognition and gait recognition (20). GCNs can effectively handle non-Euclidean data by extending the convolution paradigm from images to graphs. The human skeleton is essentially a graph-like structure connecting major points according to the natural links in the structure of human bone. Therefore, the complex architecture of the graph makes it possible to describe the dependency relationships between interconnected joints. In the medical field, GCNs have been used to classify the severity of PD based on gait assessment. Zhang et al. proposed WM-STGCN (21) that provided an effective spatio-temporal modeling method for the recognition of PD gait that outperformed the machine learning and LSTM methods. However, most existing GCN methods focus on PD detection and FoG detection, while phase detection of Parkinsonian gait and normal gait from the skeleton data has not been reported.

In summary, this study aims to boost the accuracy of gait phase recognition using ST-GCNs, meanwhile reducing the burden on data preprocessing and feature extraction, and finally to obtain accurate gait parameters for further gait-related research. Through a comprehensive comparative analysis of gait parameters between patients and healthy controls (HC), this study wish to further elucidate the differences in the performance of the deep learning model when applied to HC and PD populations. By leveraging the strong interpretability of graph-based approaches to examine the weight assignments of human skeletal points within the model, the research seeks to enhance the model's credibility and facilitate its acceptance by medical professionals.

## 2 Methods

This paper focuses on gaining insights into gait performance in Parkinson's disease through the latent features of deep graph neural networks. By directly inputting raw depth information, the model autonomously learns relevant features without the need for data preprocessing or feature engineering. This approach not only enhances the model's accuracy but also ensures its ease of deployment in clinical settings, making it broadly applicable for more precise and effective gait analysis.

### 2.1 Data set

The dataset used in this study was originally collected as part of the project “Multimodal Imaging Technology to Explore the Mechanism of Frozen Gait in Disease,” with data collection conducted at Peking Union Medical College Hospital and The Medical Review Ethics Committee approved the study (reference JS-2530). It is not publicly available because of data protection requirements.

**Figure 1:**
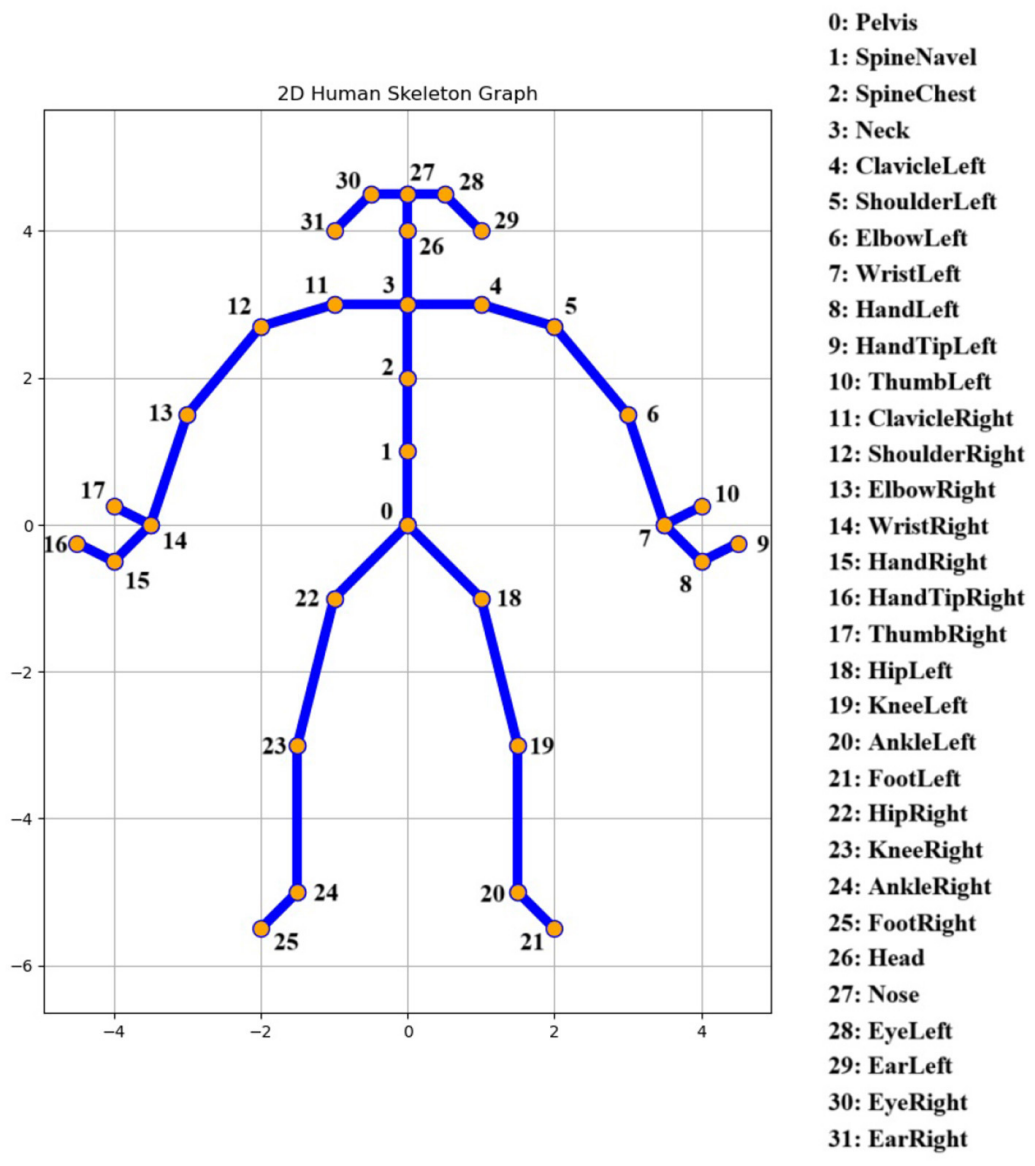
Illustration of joints and edges of human body skeleton.

The dataset includes gait performance data from 142 participants, comprising 65 individuals diagnosed with PD and 77 HC. Data was collected between 2022 and 2024 using a depth camera developed by Microsoft-Azure Kinect. In the data collection part, a 5-meter-long walking area was established in front of the Kinect. Participants were asked to walk back and forth 3 times in a natural walking posture, including actions such as standing still, walking straight forward, and turning around. Skeleton data consisting of 32 joint points was obtained from the Kinect built-in ONNX Runtime (Figure 1). The sampling frequency was 60 Hz, which was the same as the color video.

Data collection was conducted under the supervision of physicians and under local ethical guidelines, with informed consent obtained from all participants. The data used in this study were secondary analyses of previously collected research data. Participants' privacy and confidentiality were rigorously protected throughout the study. All collected data were de-identified, and any potentially identifiable information was removed before analysis.

Through preliminary interviews with clinicians, we identified concerns regarding models that solely provide PD diagnosis results. On the one hand, there are issues related to model interpretability; on the other hand, clinicians expressed uncertainty about when to trust the model and when it might make errors. Compared to features like gait speed, stride width, and gait height, clinicians are more attuned to user behavior and the timing of behavioral events. Through the interviews, we found that overlaying user behavior recognition results onto the original video interface provides a more intuitive and effective way to demonstrate the system's utility.

**Figure 2:**
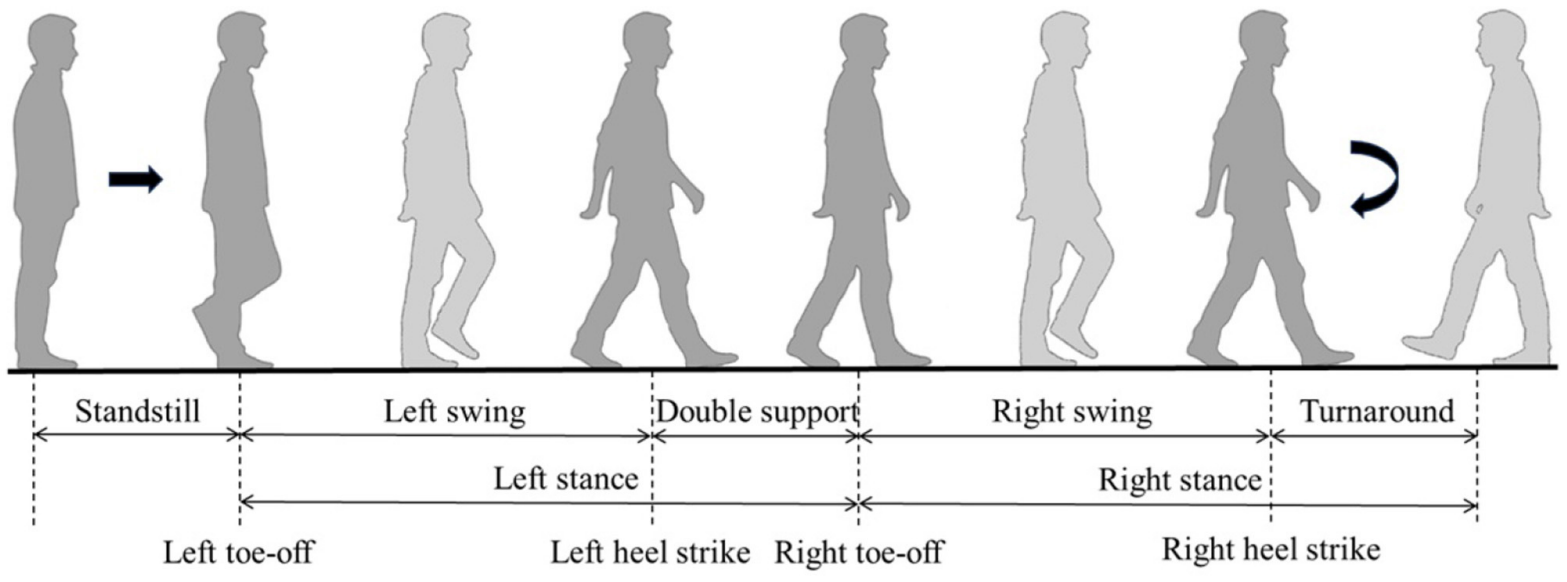
The division of gait cycle.

As a result, during the data annotation phase, we segmented gait behavior based on the presence or absence of PD, dividing the complete gait cycle into five states (Figure 2) according to the movements of the left and right feet: standstill (S), left swing (L), double support (D), right swing (R), and turnaround (T). After recording, two experts annotated the data by identifying key points of gait phase changes based on the video.

### 2.2 Deep learning models

Three different deep-learning models were trained and tested on our dataset: ST-GCN, ST-TR, and Bi-LSTM. The code repository is available at Github^1^.

Spatial-Temporal Graph Convolutional Networks (ST-GCN) (22) is a deep learning model designed for skeleton-based action recognition. It uses GCNs to model the spatial relationships between joints in a skeleton and applies temporal convolutions to capture the dynamics of these joints over time. This combination allows the model to learn both spatial and temporal features from the skeleton data, making it highly effective for action recognition tasks. The ST-GCN is composed of a set of ST-Conv blocks that alternatively apply spatial graph convolutions and temporal graph convolutions to the skeleton graph. Each ST-Conv block contains a spatial convolution layer (GCN) and a temporal convolution layer (TCN). The residual connection and bottleneck strategy are applied inside each block. The output layer contains an average pooling and a fully connected layer at the end.

The model is configured for a 5-class classification task, utilizing 3 channels (*x, y, z*), a temporal window size of 45 frame, and 32 points per frame. The window size is changable, and the 45 is best among the tests (30, 35, 40, 45). The other hyper-parameter of the training process is based on the training setting of the original ST-GCN training process. A dropout rate of 0.2 and batch normalization on data are employed for regularization and stability. The training adopts an initial learning rate of 0.01 with Nesterov momentum for enhanced convergence. Learning rate decay is scheduled at epochs 40, 80, and 120. The dataset is split into 75% training and 25% testing, with training data shuffled during each epoch. To better validate this model, we repeat the above method 4 times, as a Stratified 4-fold validation. The training employs a batch size of 64, while testing uses a batch size of 256 for efficiency. The process spans 150 epochs, applying a weight decay of 0.01 to mitigate overfitting.

Spatial-Temporal Transformer network (ST-TR) Plizzari et al. (23) is a Transformer-based model dependencies between joints using the self-attention operator. The flexibility of the Transformer self-attention in modeling long-range dependencies make models a perfect solution in Natural Language Processing (NLP) and Computer Vision (CV) tasks (24, 25). The ST-TR applys the same mechanism to joints representing the human skeleton. It uses a Spatial Self-Attention module (SSA) and a Temporal Self-Attention module (TSA) to understand intra-frame interactions between different body parts and inter-frame correlations. A two-stream architecture is used to combine the SSA and TSA modules. On the spatial stream, SSA is used to extract spatial information, followed by a 2D convolution on time dimension (TCN). On the temporal, TSA is used to extract temporal information, while spatial features are extracted by a standard graph convolution (GCN).

Bi-LSTM (26) is a variant of LSTM that captures contextual relationships in sequences by learning both forward and backward information. Bi-LSTM has achieved significant results in many fields, such as speech recognition and medicine (27), demonstrating its ability to process time series data. This allows Bi-LSTM to better understand the patterns and dependencies in a gait sequence, ultimately improving the prediction of gait labels for each frame. Recent study (18) in gait analysis indicate that this model performs better than traditional machine learning methods such as SVM.

### 2.3 Model explanation

An GCN typically consists of multiple layers, each performing graph convolutions on skeletal data across time. Formally, let *G* = (*V, E*) represent the graph, where *V* is the set of joints (nodes) and *E* denotes edges capturing joint connectivity. The network refines these representations across layers, culminating in an output layer that classifies the gait action. In GCN models, the weights associated with joints (nodes) and bones (edges) encapsulate the learned importance of each element in representing and processing the underlying data. Specifically, joint weights reflect how much individual joint features contribute to the overall representation, while edge weights modulate the influence of the relationships between joints. Higher edge weights indicate a stronger interdependency, suggesting that the connection between those joints is more critical for capturing relevant motion patterns–such as those in gait cycles. By examining these weights, one can infer which joints and interactions are prioritized by the model during decision-making, thereby linking the learned parameters to known biomechanical principles and enhancing the interpretability of the model's predictions.

### 2.4 Subjective interview

To verify the feasibility of this study's method in field medical scenarios, we conducted in-depth interviews with physicians experienced in diagnosis of Parkinson's disease. During the interviews, we collected a total of 6 conversation records from physicians. The interviews aimed to gather their insights and opinions on the results.

## 3 Results

The typical gait of PD patients includes various conditions such as unilateral lower limb tremors, bradykinesia, and postural instability. As symptoms progress, the differences in motor performance between PD patients and healthy individuals become more pronounced. In this context, the study first examines the applicability of the ST-GCN model across different populations, validating its effectiveness by recognizing five distinct activity states. We then compared the ST-GCN model with those from previous studies, discussing its scope of applicability and the potential for a similar study. Next, we applied the ST-GCN model to identify the five activity states in PD patients and HC to evaluate the model's generalizability. Finally, through interviews and discussions with clinicians, we examined the role of model parameters in enhancing model interpretability and the overall credibility of the results.

### 3.1 Comparison of models

The performance of the ST-GCN was superior to ST-TR and Bi-LSTM models in all metrics. The statistical analysis results are shown in Table 1 [the Bi-LSTM and SVM model results form Jing et al. (18)]. And the results of 4-fold validation is presented in Table 2 regarding the classification results from the five classifiers, ST-GCN demonstrated the best performance, requiring no data preprocessing or feature engineering, as it simply uses continuous raw depth images as input signals. ST-GCN achieved an average precision, recall, and F1-score of 93.48%, 93.21%, and 93.32%. The accuracy of the Bi-LSTM (93.20%) was similar to that of ST-GCN (93.73%), while other metrics were about 3% lower (90.54%, 90.41%, 90.38%). The precision, recall, and F1-score of the ST-TR were very close to those of Bi-LSTM, at 90.74%, 90.65%, 90.64%. Surprisingly, the accuracy of ST-TR (90.65%) was the lowest among those deep learning models. This discrepancy may be attributed to the smaller dataset size, fewer label categories, and lower task complexity, which may not fully leverage the capabilities of ST-TR.

**Table 1:** The performance of ST-GCN and its comparison to previous models.

**Models**	**Accuracy**	**Precision**	**Recall**	**F1-score**
SVM	0.7750	0.8699	0.8662	0.8667
Bi-LSTM	0.9320	0.9054	0.9041	0.9038
ST-TR-PD	0.9203	0.9213	0.9203	0.9213
ST-TR-HC	0.9124	0.9129	0.9124	0.9129
ST-TR	0.9065	0.9074	0.9065	0.9064
ST-GCN-PD	0.9329	0.9348	0.9321	0.9332
ST-GCN-HC	0.9386	0.9383	0.9386	0.9386
ST-GCN	0.9373	0.9382	0.9373	0.9372

**Table 2:** 4-fold validation of ST-GCN.

**Folds**	**Accuracy**	**Precision**	**Recall**	**F1-score**
Fold-1	0.9360	0.9381	0.9327	0.9353
Fold-2	0.9330	0.9372	0.9379	0.9375
Fold-3	0.9376	0.9391	0.9337	0.9363
Fold-4	0.9343	0.9367	0.9385	0.9375

### 3.2 Performance in different participants

PD patients and HC exhibit distinct gait patterns, which can impact the model's performance. Separate training allows the model to capture the unique characteristics of each group, evaluate its generalizability, and identify necessary adjustments to enhance accuracy. This approach ensures that the model is both robust and reliable for both PD patients and healthy individuals.

We evaluated the ST-GCN model using data from both the PD and HC participants, and the results are presented in Table 1. The accuracy, precision, recall, and F1-score for both groups differed by <1%.

### 3.3 Latent features interpretation

An analysis of the model's weights indicates that the joints and edges associated with the head exhibit progressively smaller weight values. This observation aligns with real-world expectations, as the spatial position of the head typically has limited relevance to the overall image context. The diminishing weights assigned to the head suggest that its contribution to the model's decision-making process is minimal, which is consistent with its reduced significance in tasks where other body regions or spatial dynamics carry greater informational value.

**Figure 3:**
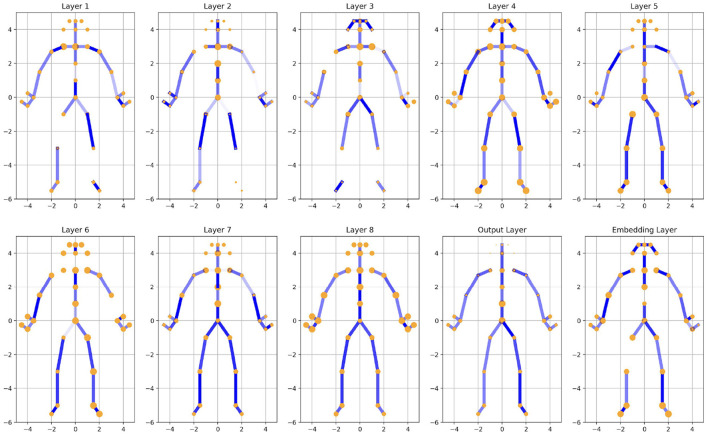
Illustration of joints and edges weights in different ST-GCN layers (PD). More transparent edges represent lower weights, and smaller joint sizes represent smaller weights.

**Figure 4:**
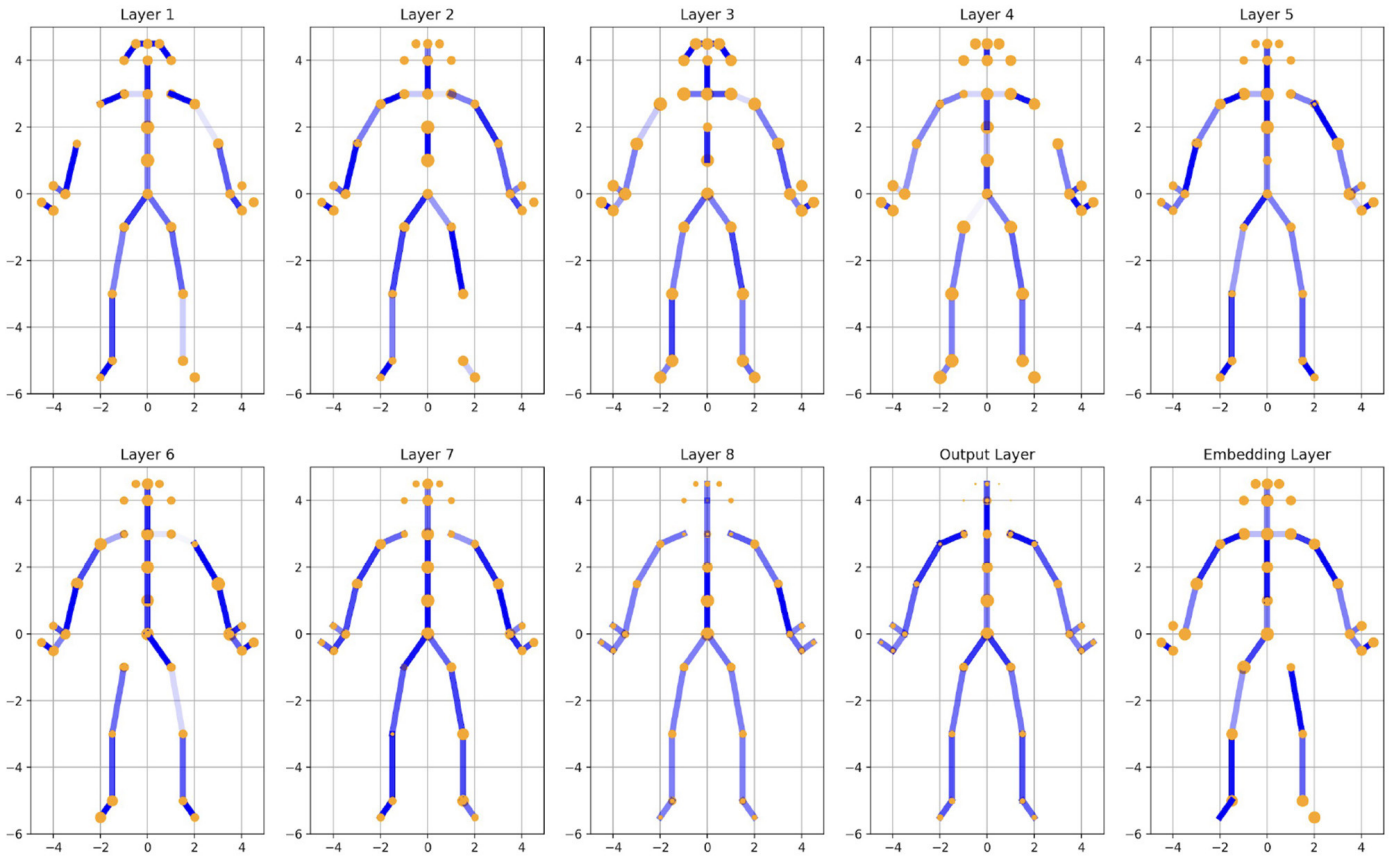
Illustration of joints and edges weights in different ST-GCN layers (HC). More transparent edges represent lower weights, and smaller joint sizes represent smaller weights.

A comparison of the two Figures 3, 4 reveals relevant distinct differences in the edge weights of the output layer between the two groups under study. In the PD group, the model weights of legs exhibit a marked asymmetry, characterized by one side being significantly lighter while the other is darker. In contrast, the model weights in the HC group display a relatively symmetrical distribution. This pronounced asymmetry observed in the PD group may be closely linked to the pathological characteristics of PD, as its onset is often unilateral, with symptoms initially manifesting predominantly on one side of the body. These findings suggest that the asymmetric weight distribution in the PD group could reflect underlying neural or behavioral asymmetries associated with the disease.

## 4 Discussion

This study aimed to apply a state-of-the-art network for spatiotemporal analysis, namely, ST-GCN for the gait analysis in skeleton data in patients with PD. This model achieved excellent accuracy in both PD patients and HC, while there were significant differences in gait performance between PD patients and healthy controls. PD patients had higher weights in what joints and what edges, which was consistent with the existing research. Due to gait being a crucial motor function in daily life, decreased gait performance is closely associated with the quality of life. Therefore, recognition and monitoring of gait is important for the diagnosis, treatment, and prognosis of PD patients. Deep learning techniques like ST-GCN make gait assessment more accurate, making PD more feasible and reliable. Furthermore, this spatiotemporal model helps to reveal potential disease characteristics of PD, thereby assisting physicians in better understanding PD.

The SVM results show significant intermittent jumps, indicating abrupt transitions between different gait states, which is inconsistent with natural human walking patterns. In interviews, clinicians noted that if they observed predicted results that were markedly different from actual user behavior and if the model's outputs were unstable, they would seriously question the system's reliability. As a result, they would be unlikely to consider such information when making diagnostic decisions.

After feature design and extraction, LSTM captures time-series information and provides reliable user behavior recognition. Because clinicians do not need to address issues such as feature selection, model fine-tuning, or adaptability to new users, they can generally allow this method.

However, clinicians interact with a large and diverse population of PD patients daily, leading to significant variability between individuals. Moreover, as PD progresses in a given patient, their motor function continues to decline, causing changes in their movement patterns. Given these factors, the performance of features used in the SVM and Bi-LSTM methods on offline datasets may not directly translate to real-world clinical diagnosis. ST-GCN, on the other hand, takes user motion data as input, allowing the model to autonomously learn motion features that capture both spatial and temporal information. This reduces the reliance on manual annotation and rule formulation, offering stronger generalization capabilities for real-world applications. Overall, the ST-GCN model's algorithm is a more suitable choice for gait assessment tasks, particularly in clinical settings.

Compared to black-box models that only provide classification results, when clinicians are shown the weight information from each layer of the ST-GCN and engage in discussions regarding the illustration of joints and edge weights across different populations (PD, HC, PD with left-foot motor impairment, and PD with right-foot motor impairment), 4 out of 6 clinicians expressed that our approach offers significant potential. This method not only helps them better understand the model's decision-making process, thereby increasing their trust in its predictions, but also mitigates biases toward artificial intelligence (AI). As a result, they are more inclined to consider the model's outputs, particularly when the model's results are presented alongside the original video without any discrepancies.

The description of the movement process has the potential to calculate additional gait parameters. However, we have not yet systematically demonstrated this capability, nor have we provided direct evidence regarding the specific improvement in accuracy offered by this method compared to traditional scale-based measurements.

## Data Availability

The datasets presented in this article are not publicly available because of data protection requirements. However, researchers interested in the data may request access by contacting the corresponding author. Requests to access the datasets should be directed to Jiecheng Wu, wujiecheng19@mails.ucas.edu.cn.

## Footnotes

^1^ https://github.com/ICEJM1020/GaitInsight
